# Impact of Nutrition Interventions on Vitamin and Mineral Intake among Native American Children and Parents: Insights from Food Resource Equity for Sustainable Health (FRESH) Study

**DOI:** 10.3390/ijerph21081014

**Published:** 2024-08-01

**Authors:** Wenjie Sun, Tori Taniguchi, Kaylee R. Clyma, Tvli S. Jacob, Valarie Blue Bird Jernigan

**Affiliations:** 1 Center for Rural Health, Center for Health Sciences, Oklahoma State University, Tulsa, OK 74107, USA; 2 Center for Indigenous Health Research and Policy, Center for Health Sciences, Oklahoma State University, Tulsa, OK 74107, USA; tori.taniguchi@okstate.edu (T.T.); kaylee.clyma@okstate.edu (K.R.C.); tvli.jacob@okstate.edu (T.S.J.); bluebird.jernigan@okstate.edu (V.B.B.J.)

**Keywords:** Native American, community-based participatory research, vitamins, minerals

## Abstract

The Food Resource Equity for Sustainable Health (FRESH) study started as a tribe community-based nutrition education program in 2018, implemented with children and parents in Osage Nation. The purpose of the FRESH study is to examine the effects of a farm-to-school family intervention on diet. The FRESH study did not directly intervene on adult caregiver participants’ diet, and, as far as we know, it is the first of its kind to implement a farm-to-school intervention in rural/tribally owned Early Childhood Education. Two communities received intervention and two served as wait-list controls. Outcomes included diet and other secondary health outcomes including vitamin and mineral intake. There were 193 children (*n* = 106 intervention; *n* = 87 control) and 170 adults (*n* = 93 intervention; *n* = 77 control) enrolled. Among adult caregiver participants, carbohydrate, cholesterol, and caffeine intake significantly decreased after the intervention (*p* < 0.05). There is a hidden hunger issue among caregivers in Native American populations. The family-based nutritional intervention, which includes educational components for caregivers, might have some effect on improving micronutrient status. Future studies focusing on key micronutrients through efficient methods are warranted.

## 1. Introduction

Native Americans encompass a diverse group of peoples. Currently, there are 574 federally recognized tribes in the United States. According to the U.S. Census Bureau, approximately 22% of the 5.2 million Native Americans in the United States live on reservations or other trust land, which is a designated area of land managed by a Native American tribe under the supervision of the U.S. government. Native American populations face high rates of diet-related health disparities, including obesity, diabetes, and hypertension. These disparities are often exacerbated in rural communities due to limited access to healthy food and high rates of food insecurity [1]. In addition, there are high rates of vitamin and mineral deficiencies within Native American populations [2]. The reasons for these disparities could be due to a lack of access to healthy foods and the effects of colonialism on traditional culture, especially regarding eating and cooking behaviors [3,4]. People from Native American groups are more likely to suffer from food insecurity compared to other populations [5,6]. Limited access to food has been associated with individuals of lower socioeconomic status, which disproportionately affects Native American populations [5]. This, in turn, is associated with the risk of insufficient intake of vitamins and minerals [7,8]. Vitamins, minerals, and other macronutrients are essential in overall health and are important to many vital organs and their functions. For example, Vitamin E has been found to have many health benefits, including its role in the prevention of disease due to its antioxidant and anti-inflammatory properties [9]. In addition, Vitamin K aids in making several compounds that are needed for many bodily functions, including blood clotting and building bones [10]. Many vitamins, like E and K, are found in vegetables that are often difficult to access in rural areas [1]. However, it is difficult to determine how to improve the vitamin and mineral status among Native American populations. While it is understood that nutritional supplementation and fortifications could be effective ways to treat vitamin and mineral deficiencies, the compliance of nutritional supplements has been found to be low, resulting in uncertainty surrounding long-term efficacy [11,12,13].

It is important to note that many Native American populations’ cultural practices are based around food cultivation, cooking, and eating, as well as long-term harmony with the environment. Historically, Native American culture surrounding food sovereignty has profoundly changed due to Westernization and industrialization [3]. Westernization and industrialization have resulted in the loss of traditional food practices for many cultures and have changed eating behaviors. Unfortunately, in other cultures, it has been found that this Westernization and industrialization, such as an increase in fast food chains, is unhealthy and cannot provide sufficient amounts of vitamins and minerals [14,15]. Moreover, the original balance of supply and demand in vitamins and minerals based on previous eating behavior has been broken due to the change in dietary patterns and consumption of food, which has, in turn, caused a series of health-related problems [14,15]. Hence, the hidden hunger might exist among Native Americans.

## 2. Materials and Methods

### 2.1. Intervention

In brief, the FRESH study, a multi-level gardening intervention, was developed by Osage Nation and university researchers. The Osage Nation reservation is situated in the northeastern region of Oklahoma. The tribe’s total membership is 11,394, with approximately 7000 residing in the reservation. The Early Childhood Education (ECE) program was conducted within the Osage Nation of Oklahoma. The tribal government is headquartered in Pawhuska, OK, and has jurisdiction over Osage County. This study took place within Osage Nation and Osage Nation ECE programs. More information about the FRESH study can be found elsewhere [16,17,18]. The FRESH study was conducted in tribally owned and operated ECE programs within the Osage Nation in Oklahoma. This multi-level intervention consisted of three main components: (1) a preschool curriculum, a 15-week nutrition and gardening curriculum at the nine ECE programs designed to increase vegetable knowledge, willingness-to-try, and taste preference; (2) a parent curriculum, a 16-week hybrid nutrition education and food sovereignty curriculum for parents/guardians, including online and in-person components; (3) ECE program menu modifications.

A total of four Osage communities were categorized into two groups: intervention (*n* = 2) and wait-listed control (*n* = 2). A total of 170 adult caregivers participated in the study. Among them, 93 participants were randomized in the intervention group, while 77 participants were randomized in the control group. The wait-listed control group received the intervention after the follow-up data collection was complete. Our organization collaborated with the Osage Nation to conduct the Food Resource Equity and Sustainability for Health (FRESH) study. This culturally relevant farm-to-school program aimed to promote vegetable consumption among Native American children and their families and was implemented in Osage Nation ECEs. The tribal–university partnership responsible for developing the FRESH study was established in 2013 and initiated by discussions between the study’s principal investigator and the Director of Communities of Excellence at Osage Nation.

The FRESH study is a randomized, wait-list controlled trial that aims to increase the consumption of vegetables and fruits among preschool-aged children and their families. The study was conducted in partnership with the Osage Nation in Oklahoma and involved a multi-level, multi-component intervention. To assign ECE programs to either the intervention or wait-list control group, we used the community as the unit of randomization. Five schools in two communities were randomly assigned to the intervention group, while the other four schools in two other communities were assigned to the wait-list control group.

Details on data collection for both children and caregivers can be found elsewhere [19]. To assess vitamin and mineral status, we used the National Cancer Institute’s Multiple Pass Automated Self-Administered 24-h Recall (ASA24) [20]. ASAs were administered to caregiver participants only and collected among both intervention and control groups at baseline and post-intervention. Recalls were obtained by trained university staff either in person or via telephone.

To the best of our knowledge, there has not been a specific family-based intervention study that has addressed hidden hunger (micronutrients) status among Native American adults in the United States. Hence, the research objective is to assess micronutrient status among adult caregivers by examining the results from the FRESH study, a farm-to-school, multi-component intervention on diet. Also, family-based nutrition intervention was evaluated by its effectiveness in improving hidden hunger. Though the FRESH study did not directly intervene in adult caregiver participants’ diet, we hypothesize that hidden hunger existed among the population, and this family-based nutritional intervention, which includes educational caregiver components, will be an effective method to improve micronutrients among caregivers in Native American populations.

### 2.2. Statistical Methods

Baseline ASA24 dietary recall characteristics were compared between the intervention and control groups. A self-control study (longitudinal comparison between pre- and post-intervention on outcomes) was performed within groups and a comparative study was performed between groups using data computed by the ASA24 dietary recall. The mean and SD (standard deviation) were used to describe the outcomes between the two groups in different stages. A Kolmogorov–Smirnov test was performed to determine the conformity of the data to the normal distribution. Some outcome variables do not strictly follow the normal distributions. However, using the central limit theorem, as the sample size increases, the distribution of the mean approaches a normal distribution.

Since the Recommended Dietary Intake (RDI) varies based on age, gender, and special needs, it is challenging to establish a uniform criterion for all participants. The parents in the current study are very young, with the mean average age of the participants at 33.2 years, excluding pregnant individuals. Although gender information is not available in this study, we conducted a simple comparison of the average RDI requirements (combining male and female) with the actual intake before and after the intervention [21].

A *t*-test was used to compare the means of the two groups, examining both baseline and post-intervention data. Also, a *t*-test was used to compare pre- and post-outcomes between the two groups on each item using ASA24 dietary recall results. SAS for Windows Statistical Software Package Version 9.4 (SAS Institute, Cary, NC, USA) was used for data processing and analysis. All the tests were two-sided, and the significance level was set at 0.05.

## 3. Results

A total of 170 caregiver participants were examined. However, only 151 had data completed for both pre- and post-intervention. The overall average age of the participants was 33.2 years. Among them, 91.8% were female, 61.6% were married, 67.3% were employed full or part-time, 40% completed high school/GED, and 71% had an annual income of USD 50,000 or less. The intervention group had a higher educational attainment and income and was employed more compared to the control group, but the difference between the two groups was nonsignificant.

Table 1 and Table 2 show no difference between the intervention and control arms regarding vitamin and mineral status prior to the intervention. However, after the intervention, there was a significantly lower intake of carbohydrates, thiamine (vitamin B1), and zinc among the intervention group compared to the control arm (*p* < 0.05). Post-intervention, carbohydrates, zinc, and thiamine were 166.0 g. (SD = 94.7), 8.4 mg. (SD = 4.8), and 1.1 mg. (SD = 0.8), respectively, while those in the control group were 105.4 g. (SD = 147.4), 10.3 mg. (SD = 6.3), and 1.3 mg (SD = 0.8), respectively. In addition, a self-comparison shows that among the intervention group, lutein and zeaxanthin and Vitamin K significantly increased, but carbohydrates, caffeine, cholesterol, and theobromines significantly decreased after the intervention (*p* < 0.05). The control group showed a significant difference regarding carbohydrates and caffeine intake post-intervention compared to baseline, as shown in Table 3.

The RDI criteria are significantly higher than the average intake of the adult participants, both before and after the intervention, in both the intervention and control groups, including micronutrients (*p* < 0.05), such as vitamins A, C, and E, as well as minerals like calcium and magnesium, but not macronutrients (except fiber) (*p* > 0.05). For instance, the RDI of vitamin A is 900 μg for males and 700 μg for females, while the measured intake of vitamin A is much lower. Similar discrepancies were observed for other vitamins and minerals. The criteria for these vitamins and minerals, according to the RDI, are listed in the attachment. This suggests that there may be a hidden hunger issue, which is defined as the non-explicit need for one or more of the 26 micronutrients essential for adequate body function among the Native American participants. Although the intervention group still did not meet the RDI criteria for vitamins and minerals after the intervention, the difference between the RDI and actual intake was reduced.

Regarding macronutrients, although they already meet the RDI requirements, it indicates that the Native American participants may be experiencing potential malnutrition due to food insecurity. (Table 3 and attachment).

Our study found no significant differences in the intake of most vitamins and minerals before and after the intervention when compared to RDI values. However, the significant behavioral changes observed, such as the increased consumption of targeted vegetables and decreased intake of certain macronutrients, suggest that the intervention had positive dietary effects that may contribute to long-term health improvements, although those are much lower than the RDI criteria. These findings indicate that while immediate changes in micronutrient intake were minimal, the intervention successfully promoted healthier eating behaviors, which could lead to better nutritional outcomes over time.

## 4. Discussion

The results show that there was a significantly lower intake of thiamine and zinc in the intervention group than in the control group. In addition, a self-comparison shows that among the intervention arm, zeaxanthin and vitamin K, significantly increased, while carbohydrates, caffeine, Cholesterol, and Theobromine significantly decreased after the intervention.

Iron and zinc deficiencies are commonly found in those with plant-based diets [22]. Like results from previous studies, the FRESH study shows a significantly lower vitamin B1 status in vegetarians than in non-vegetarians [23]. Hence, an increase in the consumption of vegetables in the intervention arm could partly explain the significantly lower status of zinc and vitamin B1 post-intervention compared with the control arm. Other vitamins and minerals might have multiple intake channels from foods. The vegetables in the control arm increased by about 20% compared with baseline. A possible explanation for this is that the primary food sources of zinc are meat, fish, and seafood, whereas vitamin B1 is primarily found in pork, fish, and other sources, although some plant-based foods like green peas, enriched cereals, breads, noodles, and rice also contain vitamin B1. When the percentage of vegetables in a meal increases, the relative proportion of other food components may decrease if the total amount of food is held constant. According to the National Health and Nutrition Examination Survey (NHNES), there is a huge percentage of the U.S. population that uses dietary supplements, which is also an indirect indicator of the disadvantages of Westernization and industrialization on food [24]. Caffeine intake was also measured, and intake significantly decreased. After the intervention, the total consumption of caffeine decreased from 203.6 mg. to 166.0 mg., which is a significant difference when compared to the consumption of 197.5 mg in the control group at post-intervention. A possible explanation is that the participants changed their food selection, increasing the intake of vitamins. As we know, coffee, which is a common source of caffeine, may have negative health effects. For instance, caffeine can cause blood pressure variation, have a negative effect on diastolic blood pressure, and contribute to hypertension [25]. Even though caffeine has been found to be genotoxic at high levels of concentration [26], for some Native American populations, caffeine in its liquid form is traditionally consumed for use in ceremonies and rituals [27]. This suggests that the FRESH study, a family-based nutritional intervention, can change Native Americans’ eating behaviors and result in decreased intake of coffee.

One possible explanation for the high vitamin K level and low cholesterol level in the intervention group could be due to the increased vegetable consumption among those participants. As we know, vegetables, especially leafy greens, contain vitamin K [28]. High vitamin K intake can support reducing body weight, as well as abdominal and visceral fat levels [29]. Although the association between cholesterol levels and health outcomes is unclear, our results are in line with previous nutritional intervention studies, concluding that it can be an efficient and effective way to reduce cholesterol levels compared to medical interventions [30,31,32].

We compared the intake of vitamins and minerals against the RDI values for each nutrient. The comparison revealed that, despite the intervention, most participants’ intake levels remained below the RDI for several key vitamins and minerals, such as Vitamin D, Calcium, and Iron. These findings highlight the persistent nutritional gaps in the population studied and underscore the need for more intensive or prolonged interventions.

Regarding macronutrients, although they already meet the RDI requirements, it indicates that the Native American participants may be experiencing potential malnutrition due to food insecurity. Therefore, nutrition interventions are warranted.

Our study had a public health implication. To the best of our knowledge, this is the first community-based intervention study addressing vitamin and mineral intake within Native American populations in the United States. It provides useful information for Native American populations and related health policy makers.

These findings highlight the importance of a well-balanced diet rich in essential vitamins and minerals for the prevention and management of diet-related health disparities. Our study suggests that targeted nutritional interventions focusing on these micronutrients can be an effective strategy in mitigating the adverse effects of nutritional deficiencies and improving overall metabolic health. By providing a comprehensive analysis of the intake levels, we aim to highlight the potential benefits of adequate vitamin and mineral consumption as part of an integrated approach to improving health outcomes. Future research should continue to explore these relationships to develop precise dietary recommendations for Native American populations, address the potential malnutrition due to food insecurity, and strive to meet RDI criteria.

### Limitations and Strengths

Our study has several limitations. The study design cannot allow us to conduct randomized trials in the community. The population of this Native American community provided a small sampling size within the geographic location, making it difficult to separate individuals in the control and intervention groups and potentially providing the opportunity for overlap between communities that are receiving the intervention and those in control. For future studies, a suggestion would be to recruit from similar Indigenous communities in order to obtain a larger sample size. To see significant change in behavior, the study must take place over a longer period of time in order for it to take effect. Because the intervention period was only five months, it is difficult to evaluate long-term effects.

It is also difficult to compare the change in vitamin and mineral status using a child-based nutritional intervention given that data were only collected at baseline and post-intervention on caregiver participants using a self-reported dietary recall, the ASA24. ASA24 data could be either an overestimation or an underestimation of the actual vitamin and mineral status. Due to the nature of the intervention, vitamin intake varied due to which vegetable was being consumed at the time, making a pre- and post-intervention measurement less accurate. It is necessary to repeat this measure throughout the intervention to provide a more concrete conclusion regarding the change in nutritional status. Hence, a study with a larger sample size and a longer intervention period is warranted.

Though the results from the presented study provide evidence to address key determinants of health, including food/nutrition insecurity among the Native American population, more evidence is needed to inform both tribal and federal policy makers and programs. Our results indicate that there is a hidden hunger issue among caregivers in Native American populations. The family-based nutritional intervention, which includes educational components for caregivers, might have some effect on improving micronutrient status. Future studies focusing on key micronutrients through efficient methods are warranted.

## Figures and Tables

**Table 1:** Mean and Standard Deviation of Minerals Pre- and Post-Intervention.

	Baseline (*n* = 170)		Post-Intervention (*n* = 151)	
	Intervention (*n* = 93)	Control (*n* = 77)	Intervention (*n* = 85)	Control (*n* = 66)
Minerals				
Calcium (mg)	826 ± 556	853 ± 533	781 ± 461	874 ± 482
Iron (mg)	11 ± 7	11 ± 5	10 ± 6	12 ± 7
Magnesium (mg)	216 ± 99	224 ± 93	205 ± 111	240 ± 107
Phosphorus (mg)	1103 ± 514	1177 ± 601	1079 ± 559	1201 ± 580
Potassium (mg)	1987 ± 947	1981 ± 843	1756 ± 881	2026 ± 910
Sodium (mg)	3081 ± 1379	3111 ± 1340	2816 ± 1442	3084 ± 1488
Zinc (mg)	9 ± 5	9 ± 5	8 ± 5 §	10 ± 6
Copper (mg)	1 ± 1	1 ± 0	1 ± 1	1 ± 1
Selenium (mcg)	92 ± 46	90 ± 49	86 ± 49	101 ± 54

* Comparison between pre- and post-intervention in the same group (*p* < 0.05). § Comparison between intervention and control group at same stage (*p* < 0.05).

**Table 2:** Mean and Standard Deviation of Vitamins Pre- and Post-Intervention.

	Baseline (*n* = 170)		Post-Intervention (*n* = 151)	
	Intervention (*n* = 93)	Control (*n* = 77)	Intervention (*n* = 85)	Control (*n* = 66)
Vitamins				
Vitamin C (mg)	55 ± 93	45 ± 52	41 ± 43	52 ± 51
Thiamine (mg)	1 ± 1	1 ± 1	1 ± 1 §	1 ± 1
Riboflavin (mg)	2 ± 1	2 ± 1	1 ± 1	2 ± 1
Niacin (mg)	20 ± 10	19 ± 10	18 ± 11	21 ± 13
Vitamin B6 (mg)	2 ± 2	2 ± 1	2 ± 1	2 ± 2
Folate (mcg)	290 ± 191	287 ± 148	283 ± 213	316 ± 227
Folic Acid (mcg)	144 ± 170	125 ± 85	134 ± 170	150 ± 193
Folate, food (mcg)	146 ± 82	162 ± 107	150 ± 122	166 ± 96
Foldate, DFE (mcg_DFE)	391 ± 303	375 ± 194	377 ± 318	421 ± 354
Vitamin B-12 (mcg)	3 ± 3	4 ± 5	4 ± 5	4 ± 3
Vitamin A, RAE (mcg_RAE)	440 ± 357	474 ± 373	390 ± 330	490 ± 367
Retinol (mcg)	301 ± 283	357 ± 315	258 ± 210	324 ± 289
Carotene, beta (mcg)	1453 ± 2671	1243 ± 1734	1480 ± 2232	1784 ± 2746
Carotene, alpha (mcg)	364 ± 860	266 ± 562	180 ± 562	362 ± 980
Cryptoxanthin, beta (mcg)	59 ± 118	52 ± 101	49 ± 98	53 ± 101
Lycopene (mcg)	4881 ± 7223	4652 ± 6382	3993 ± 7198	3474 ± 4593
Lutein + zeaxanthin (mcg)	861 ± 975	1081 ± 1767	1728 ± 2926 *	1206 ± 1718
Vitamin E, alpha-tocopherol (mg)	6 ± 4	6 ± 4	6 ± 5	7 ± 5
Vitamin K, phylloquinone (mcg)	75 ± 69	87 ± 106	105 ± 120 *	91 ± 116
Cholesterol (mg)	264 ± 194	274 ± 257	210 ± 176 *	247 ± 215
Fatty acids, total saturated (g)	23 ± 14	24 ± 15	21 ± 14	22 ± 13

* Comparison between pre- and post-intervention in the same group (*p* < 0.05). § Comparison between intervention and control group at same stage (*p* < 0.05).

**Table 3:** Mean and Standard Deviation of Macronutrients Pre- and Post-Intervention.

	Baseline (*n* = 170)		Post-Intervention (*n* = 151)	
	Intervention (*n* = 93)	Control (*n* = 77)	Intervention (*n* = 85)	Control (*n* = 66)
Macronutrients				
Protein (g)	68 ± 29	66 ± 34	66 ± 37	75 ± 38
Total fat (g)	69 ± 35	69 ± 38	61 ± 37	65 ± 32
Carbohydrate (g)	204 ± 112 §	244 ± 116	166 ± 95 * §	198 ± 106 *
Water (g)	2650 ± 1311	3079 ± 1823	2271 ± 1503	2652 ± 1287
Alcohol (g)	2 ± 11	2 ± 7	3 ± 16	1 ± 4
Caffeine (mg)	145 ± 148	167 ± 162	98 ± 119 *	105 ± 147 *
Theobromine (mg)	34 ± 67	42 ± 49	22 ± 48	23 ± 50 *
Sugars (g)	99 ± 77 §	131 ± 88	75 ± 60 *	92 ± 70 *
Fiber, total dietary (g)	12 ± 7	12 ± 7	11 ± 8	13 ± 8

* Comparison between pre- and post-intervention in the same group (*p* < 0.05). § Comparison between intervention and control group at same stage (*p* < 0.05).

## Data Availability

The de-identified data presented in this study are available on reasonable request from the corresponding authors and can only be released following permission from the senior author and Osage Nation. The data are not publicly available in order to protect the privacy of the participants.
